# Improvement of Seed-Mediated Growth of Gold Nanoparticle Labels for DNA Membrane-Based Assays

**DOI:** 10.3390/bios13010002

**Published:** 2022-12-21

**Authors:** Galina V. Presnova, Gleb A. Zhdanov, Luibov Yu. Filatova, Mariya M. Ulyashova, Denis E. Presnov, Maya Yu. Rubtsova

**Affiliations:** 1 Department of Chemistry, M.V. Lomonosov Moscow State University, 119991 Moscow, Russia; 2 D.V. Skobeltsyn Institute of Nuclear Physics, M.V. Lomonosov Moscow State University, 119991 Moscow, Russia; 3 MSU Quantum Technology Centre, 119991 Moscow, Russia; 4 Cryoelectronics Lab, Faculty of Physics, M.V. Lomonosov Moscow State University, 119991 Moscow, Russia

**Keywords:** gold nanoparticles, seed-mediated growth, staining enhancement, membrane-based assays, DNA hybridization

## Abstract

Gold nanoparticles (AuNPs) are popular labels for colorimetric detection of various analytes, involving proteins, nucleic acids, viruses, and whole cells because of their outstanding optical properties, inertness, and modification variability. In this work, we present an improved approach for enhancement of color intensity for DNA membrane microarrays based on seed-mediated growth of AuNP labels. Biotin-labeled DNA is hybridized with capture oligonucleotide probes immobilized on the microarrays. Then biotin is revealed by a streptavidin–AuNP conjugate followed by the detection of AuNPs. Optimization of seed-mediated enlargement of AuNPs by the reduction of tetrachloroauric acid with hydroxylamine made it possible to change the coloring of specific spots on the microarrays from pink to a more contrasting black with minor background staining. Mean size of the resulting AuNPs was four times larger than before the enhancement. Adjusting the pH of HAuCl_4_ solution to 3.5 and use of a large excess of hydroxylamine increased the signal/background ratio by several times. The method’s applicability was demonstrated for quantification of a short oligonucleotide of 19 bases and full-length TEM-type β-lactamase genes of 860 bp responsible for the development of bacterial resistance against β-lactam antibiotics. Improved protocol for AuNP enlargement may be further transferred to any other membrane-based assays of nucleic acids with both instrumental and visual colorimetric detection.

## 1. Introduction

Active development of nanotechnology and new nanomaterials has resulted in various novel biosensors with nanoparticles as a label for determination of different biological objects including proteins, nucleic acids, bacteria and viruses [1,2,3]. To date, a variety of detection techniques have been applied for biosensors: colorimetric [4], fluorescent [5], magnetic [6], electrochemical [7], surface enhanced Raman scattering [8,9] and plasmonic resonance [10,11]. Among them, colorimetric membrane-based assays such as Lateral Flow Immunochromatographic Assay (LFIA) and microarrays are attractive in implementation because test line or spot coloration can be assessed visually by naked-eyes or quantitatively by scanometric detection using inexpensive flatbed scanners [2,12].

Gold nanoparticles (AuNPs) have outstanding optical characteristics such as a high extinction coefficient and a size-dependent surface plasmon resonance (SPR) band position [13,14,15]. Despite these advantages of AuNPs as labels for colorimetric detection, they may lack sensitivity for biosensing purposes due to their low density in the location of interest and pale visible staining. It was revealed that AuNPs smaller than 15 nm in diameter cannot produce an intense color, while nanoparticles larger than 60–70 nm are unstable for conjugation [16]. This is why additional stages of signal amplification are needed. Different amplification strategies have been suggested based on the aggregation of nanoparticles or their enlargement due to reduction of silver or gold salts [16,17,18,19]. Catalytic deposition of newly synthesized NPs onto existing nanoparticles as seeds enlarges them, and their molar extinction coefficient increases [20]. As a result, the test spot becomes more darkly colored and contrasting. This approach is regarded as quite promising because of the increase in size of nanoparticles after analyte recognition and because it does not promote steric hindrance for the binding of analytes with capture molecules.

Silver enhancement has been actively applied for the determination of different analytes (toxins, bacterial antigens, viruses, nucleic acids) by LFIA [21,22,23,24]. A decrease in detection limits has been reported due to the formation of dark gray nanoparticles, which are more contrasting in comparison to pink or red ones. At the same time, application of this approach revealed obvious disadvantages associated with self-nucleation, non-specific staining, and irreversible precipitation with chloride and phosphate ions. Gold enhancement of AuNPs using tetrachloroaurate (III) reduction was shown to avoid the abovementioned disadvantages [25]. The color of enlarged nanoparticles changes to dark purple depending on their size due to the plasmon coupling effect of neighboring particles [26] and local changes in dielectric permittivity [27,28]. To reduce Au^3+^ ions, it was proposed to use various reducing agents (sodium borohydride, ascorbic acid, peroxides, hydroxylamine) [29,30,31,32]. Mild reduction with hydroxylamine promotes the growth of seeds and lack of nucleation, resulting in reduced non-specific staining. An increase in the sensitivity of LFIA has been demonstrated for various analytes (cells [33], proteins [34], ions [35], and nucleic acids [36]).

In recent years, gold enhancement has been used to improve the sensitivity of nucleic acid identification on glass microarrays [37]. We believe that this method is also promising for increasing the sensitivity of DNA determination by membrane-based assays, including scanometric microarrays. The effect of AuNP amplification conditions by gold ion reduction has been studied in several works [38,39,40], but it has not been examined in detail for porous membranes. This work aims at optimizing the conditions for seed-mediated growth of AuNP labels of DNA duplexes formed on nitrocellulose membranes. Hydroxylamine was chosen as the reducing agent for tetrachloroauric (III) anions. First of all, we studied the effect of pH, since a local change in the pH of charged nucleic acid molecules on a charged polymer surface can affect the growth of seeds and nucleation. We also optimized the ratios of the reagents and attributed them to the shapes and sizes of the enlarged AuNPs in order to increase signal-to-noise ratios and assay sensitivity. Applicability of this technique was demonstrated for determination of short single-strand 19-mer oligonucleotide and long double-strand DNA. The full-size genes (860 bp) of the serine TEM-type β-lactamases (four variants) were used as examples of long DNAs. Determination of β-lactamase genes is of great practical importance as they confer bacterial resistance to β-lactam antibiotics [41].

## 2. Materials and Methods

### 2.1. Materials

Tetrachloroauric acid, hydroxylamine, mercaptosuccinic acid, dithiothreitol, ethylenediaminetetraacetic acid (EDTA), sodium dodecyl sulfate (SDS), N-hydroxysuccinimide (NHS), 3-(3-dimethylaminopropyl)-1-ethyl-carbodiimide hydrochloride (EDC), bovine serum albumin (BSA), casein, Tween-20, were purchased from Sigma-Aldrich (St. Louis, MO, USA); streptavidin was obtained from Imtek (Moscow, Russia); dNTPs, Taq DNA polymerase, and dUTP-11-Biotin were obtained from Fermentas (St. Leon-Rot, Germany); acids, alkali, salts and solvents were purchased from Chimmed (Moscow, Russia). All chemicals were of analytical grade. All water used in experiments was purified with a Milli-Q system (Millipore, Billerica, MA, USA).

Primers, capture oligonucleotide probes containing a 5′-amino group and 13-mer thymidine spacer and target oligonucleotide of 19 bases containing a 5′-biotin were synthesized and purified by Syntol (Moscow, Russia). Their sequences are listed in Table S1. Sequence of model target oligonucleotide was complementary to the capture oligonucleotide probe.

Samples of full-size TEM-type β-lactamase genes were obtained from total DNA fractions extracted from laboratory strain *E. coli*—producer of recombinant β-lactamase TEM-1 and from four clinical laboratory strains resistant to β-lactam antibiotics (*E. coli* J53, harboring β-lactamase TEM-1; *E. coli* I53, harboring β-lactamase TEM-3; *E. coli* 190, harboring β-lactamase TEM-9; *E. coli* 199, harboring β-lactamase TEM-37 (IRT 8)), which were kindly provided by Dr. M.V. Edelstein (Institute of Antimicrobial Chemotherapy of Smolensk State Medical Academy, Smolensk, Russia). DNA fractions were obtained with the InstaGene Matrix Kit (Bio-Rad, Hercules, CA, USA) and used as a template for PCR amplification. Target DNAs of TEM-type β-lactamases were amplified by the PCR and labeled with biotin by introducing dUTP-11-biotin as described in [42].

### 2.2. Synthesis and Characterization of Gold Nanoparticles and Their Conjugate with Streptavidin

AuNPs with a diameter of 33 ± 2 nm were prepared by reduction of tetrachloroauric acid with sodium citrate [43]. For this, 50 mL of an aqueous 0.01% tetrachloroauric acid was heated to boiling in an Erlenmeyer flask with a reflux condenser, then 0.5 mL of aqueous 1% sodium citrate was quickly added with stirring and boiled for another 15 min, continuing stirring. AuNPs size was determined by scanning electron microscopy (SEM) on Supra-40 microscope (Carl Zeiss, Germany) equipped with an InLens secondary electron detector built in the microscope column (Figure S1). A conjugate of streptavidin with AuNPs was prepared by covalent attachment of AuNPs to streptavidin modified with mercaptosuccinic acid as described earlier [44].

The ζ–potentials of the samples were measured using a Zetasizer Nano ZS analyzer (Malvern, UK, He–Ne laser, 5 mW, 633 nm) with a detection angle of 13°. The measurements were carried out at 25 °C in U–shaped clear disposable zeta cells (with integrated gold electrodes). All obtained ζ–potential values were expressed as a mean ± SD from three independent measurements.

The AuNPs attached to DNA duplexes on membrane support were detected by SEM at relatively high accelerating voltage (20 kV) in order to achieve the best contrast and resolution. Under these conditions, the membranes are almost transparent to the electron beam, which makes it possible to avoid charge effects during the imaging of the AuNPs.

### 2.3. Membrane Microarray Fabrication

Pre-activation of BioTrace NT nitrocellulose membranes of 0.45 m pore size (PALL, www.pall.com (accessed on 15 December 2021)) was performed as follows: the membranes were acidified with 0.1 M HCl (5 min), then they were incubated in a solution containing 1% EDC and 0.2% NHS at room temperature for 30 min, followed by rinse with distilled water for 1 min. Spotting of the capture and control oligonucleotide probes and blocking of the membranes were performed as described earlier [45].

### 2.4. Hybridization of Target DNA on Microarrays

The membranes were placed in the wells of a 48-well polystyrene plate (Greiner Bio-One, Kremsmünster, Austria, www.greiner.com (accessed on 15 December 2021), hybridized with model target oligonucleotide or target DNA and then incubated in a solution of the streptavidin–AuNPs conjugate as described in [45] without additional fragmentation of the long DNA.

### 2.5. Scanometric Detection and Data Processing

The membranes were scanned with a scanner Perfection V750 Pro (Epson, Germany, https://epson.com (accessed on 15 December 2021) at a resolution of 4800 dpi. The obtained images in the TIFF format were quantitatively processed with the Scan Array Express software (PerkinElmer, version 3.0, Rodgau, Germany). The limit of detection (LOD) of target oligonucleotide or target DNA was calculated as the mean signal intensity registered for a blank sample (0 pM DNA) plus two standard deviations (SD; *n* = 10).

### 2.6. Signal Amplification

The enhancement solution consisted of aqueous solutions of tetrachloroauric acid and hydroxylamine at various concentrations (HAuCl_4_, from 0.15 to 1.50 mM; NH_2_OH, from 5 to 75 mM). The pH of the tetrachloroauric acid solution was preliminarily adjusted to a certain value with 0.1 M HCl. The solutions of two components were quickly mixed to obtain an enhancer solution and the membranes were then immersed in it. After incubation for 2 min, the staining intensities of the spots and areas around them were determined as described above.

## 3. Results and Discussion

### 3.1. Principle of Determining Nucleic Acids on Membrane-Based Microarrays with Signal Amplification

**Figure 1:**
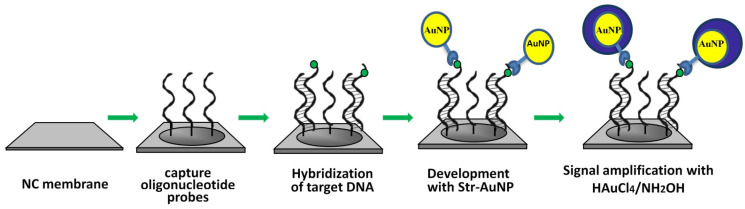
Scheme of successive stages of target oligonucleotide hybridization on membrane-based DNA-microarrays followed by seed-mediated growth of AuNP labels.

The principle of nucleic acid determination is based on the hybridization of target DNA labeled with biotin with capture probes immobilized on the surface of a nitrocellulose membrane (Figure 1). Nitrocellulose is preliminary activated by EDC and NHS. Capture (complementary) and control (non-complementary) oligonucleotide probes with a 13 T spacer and an amino-group at the 5′-end are covalently immobilized on the membrane in several repetitions in the form of a microarray. Biotin-labeled DNA is hybridized on the microarrays, and the biotin labels in DNA duplexes are developed by a conjugate of streptavidin with AuNPs of 33 nm in diameter. Then, after washing, the membranes are moistened with an enhancing solution in order to increase the size of the AuNPs. Next, the staining intensity of the microarray spots is determined. The appearance of a colored spot on the membrane confirms the presence of the DNA of interest. Staining of the spot with a non-complementary probe indicates non-specific target binding.

To ensure post-assay AuNP growth, we used the reduction of gold from a solution of tetrachloroaurate (III) by hydroxylamine as a mild reducing agent. The reduction can take place in two ways: (i) AuNP labels with excess electron density on the surface serve as seeds and, as a result, increase in size, and (ii) the formation of new seeds in a solution. It is well known that bigger spherical AuNPs scatter light stronger than smaller [46]. To increase the sensitivity of the assay and preserve its specificity, one is interested in the predominant reaction on the surface of AuNP labels, since the formation of new seeds will lead to background staining. When using this process for analytical purposes, it is also important to avoid rapid aggregation and coagulation of nanoparticles. The ratio of parallel particles’ enlargement, nucleation and aggregation depends on reaction conditions, primarily on the pH of the seed solution. HAuCl_4_ is capable of exchanging a proton: it is the most oxidative in a form of (AuCl_4_)^−^ at low pH and the least reactive at high pH values [47]. Hydroxylamine is characterized by pKa = 5.2 [38]; so it presents in a solution in a protonated form (NH_3_OH)^+^ at pH below 5.2 and in a deprotonated form (NH_2_OH) at higher pH values.

### 3.2. Effect of pH on the AuNP Growth during Au^3+^ Reduction in Solution

**Figure 2:**
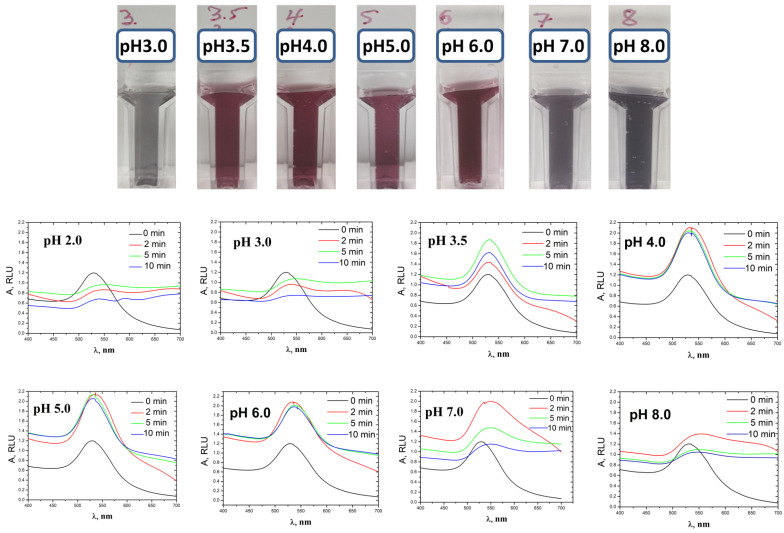
Spectra of colloidal suspensions of gold nanoparticles during the reduction of HAuCl_4_ with NH_2_OH at different pH and reaction time. The black lines correspond to the spectra of the colloidal suspensions without amplification.

The impact of pH on the growth of native AuNPs and AuNPs in the conjugate with streptavidin was first studied in a solution, and then on membrane surface. Changes in the optical properties of colloidal suspensions of AuNPs were studied by spectroscopy in the visible region (Figure 2), which makes it possible to monitor the growth of the nanoparticle size by the shift of the absorption peak, its broadening, and the changes in maximal and integral intensities. The characteristics of the spectra are given in Table S2.

When the reaction was carried out at a highly acidic pH (from 2.0 to 3.0), the absorption intensity sharply decreased as early as 2 min after the start of the reaction. The color of the solution changed from purple to gray, corresponding to a shift in the absorption maximum to the longwave region. At the same time, the maximum and integral signal intensities decreased compared to original data without amplification. This indicates the absence of AuNP seeding and the rapid aggregation and precipitation of nanoparticles newly synthesized.

At pH 3.5, the intensity of the adsorption peak increased during the first five minutes. Then the maximum intensity of the peak at 533 nm began to decrease, while a shoulder appeared on the spectrum, indicating the synthesis of a small number of larger particles. Since there were few of these particles, the overall color of the solution did not change (dark pink), and the suspension remained stable.

At acidic pH (from 4.0 to 5.0), a rapid increase in the adsorption maximum was observed at the start of the reaction, which then did not depend on time. The color of the solution became a little saturated; the intensity peak shifted towards a slight increase in the size of the nanoparticles, and the integral intensity slightly decreased with reaction time. These data are in good agreement with previous data [25,48], which show that the growth of nanoparticles and the reduction of Au^3+^ occurs in the first 5 s, and then the intensity does not change. This indicates that no particle aggregation, but also no significant amplification of the particle size, was observed in this pH range.

At slightly acidic pH from 5.0 to 6.0, the right shoulder of the spectrum broadened i.e., the size of the nanoparticles increased. The color of the solution turned purple, and both the peak intensity and the integrated intensity increased. The suspension of the nanoparticles remained stable throughout the reaction and coagulation of the nanoparticles was not observed.

At neutral pH, a smooth strong shift of the plasmon resonance peak was observed; it also significantly broadened. The maximum and integral intensity of the spectrum increased significantly at the beginning of the reaction and then slowly decreased. Color of the suspension changed to gray. This means an increase in the size of nanoparticles while the suspension of nanoparticles remains stable. The broadening of the peak can also be associated with uneven growth of the nanoparticle size.

At alkaline pH (8.0), the shift in the plasmon resonance peak was less noticeable; the color of the solution changed from purple to gray. The particles became larger (strong shift in the absorption maximum) and some of them precipitated slowly compared to fast precipitation of most of the nanoparticles at a highly acidic pH; as a result the integral intensity decreased.

It can be concluded that the processes of increasing the size of nanoparticles and the formation of new nuclei depend on the protonation of hydroxylamine, which is determined by its pKa value. At pH values close to the pKa value, the increase in the size of the nanoparticles occurs slowly and insignificantly, while the suspension remains stable. The increase in the size of existing nanoparticles as seeds is the more intensive, the farther the pH value is from the pKa value. At acidic pH, (NH_3_OH)^+^ prevails in the solution, size growth is slow, and the coagulation of nanoparticles increases up to precipitation. At pH above 6.0, hydroxylamine is deprotonated and becomes a stronger reducer for gold ions compared to the protonated form while the suspension of nanoparticles is more stable. Therefore, it is optimal to perform seed-mediated growth of AuNPs in a solution at a pH range from 6.0 to 7.0.

### 3.3. Effect of pH on the Growth of AuNP in a Conjugate with Streptavidin during Au^3+^ Reduction in Solution

**Figure 3:**
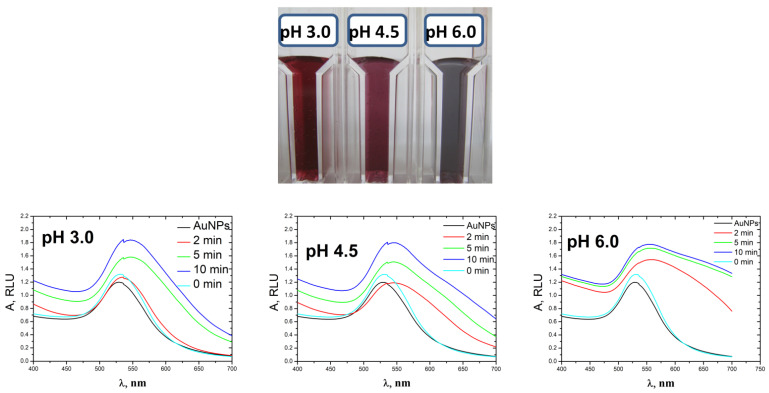
Spectra of streptavidin-AuNP conjugate during the reduction of HAuCl_4_ with NH_2_OH at different pH and reaction time. The black lines correspond to the spectra of colloidal solutions without amplification.

Next, we studied the increase in the size of AuNPs in the conjugate with streptavidin, since this is the one used in DNA analysis (Figure 3). The characteristics of the spectra are given in Table S3. Similar effects (increase and shift of the plasmon resonance peak) were observed for growth of AuNPs covalently attached to streptavidin but they occurred at lower pH values compared to native AuNPs. At pH 3.0, the shift of the peak stopped after 5 min of amplification; the integral intensity continued to increase slowly, most likely due to the coarsening of the nanoparticles. With an increase in pH to 4.5, the rate of gold reduction on the surface of nanoparticles with streptavidin increased and reached its maximum value. At pH 6.0, the plasmon resonance peak shifted to the long wavelength region quite quickly; its intensity was maximal, and the color of the suspension changed to violet. In this case, the suspension of the nanoparticles was stable; no aggregation was observed. At neutral and weakly alkaline pH, particles enlarged in size and then did not change. The broadening of the right shoulder of the spectrum indicates the non-homogeneity of the resulting nanoparticles. In general, suspensions of nanoparticles based on the conjugate are more stable; an increase in the diameter of nanoparticles can reach larger sizes compared to native AuNPs. It can be assumed that the streptavidin molecule, which partially covers the surface of the nanoparticle, is involved in these processes.

The stability of nanoparticles in a suspension is determined by electrostatic interactions with the outer layer of counter ions and particle size. The redistribution of ions in an aqueous medium can significantly impair the resistance of nanoparticles to aggregation. To characterize the stability of suspensions of native nanoparticles and the conjugate, we determined the values of their ζ-potentials at different pH (Table 1). According to [49], gold nanostructures turn out to be unstable and aggregate at absolute values of the ξ-potential less than 30 mV and they are stable at higher values of this parameter in modulus. Thus, the suspension of gold nanoparticles is unstable at pH lower than 4.5 due to coagulation and should be stable at pH above 4.5. The partial precipitation of nanoparticles observed in our experiment at pH above 8.0 (Figure 2) is most likely caused by large heterogeneity of the suspension: many large nanoparticles are formed, some of which can aggregate and precipitate but this process occurs slowly.

AuNPs in the conjugate with streptavidin are characterized by the ζ-potential values lower in modulus over the pH range from 4.5 to 7.5 compared to native nanoparticles. The obtained values indicate that the solution of AuNPs with streptavidin can be stable only at alkaline pH exceeding 7.5. However, in the experiment we observed the formation of stable suspensions of streptavidin-AuNPs conjugate in a wide range of pH from 3.0 to 6.0 (Figure 3). Apparently, charged streptavidin molecules, which partially cover the surface of nanoparticles, play a stabilizing role. A change in the total charge of the streptavidin at pH above 5.0 causes a significant change in the value of the ζ-potential and prevents aggregation. At pH 6.0, suspension is also stable, despite the significant heterogeneity of particles in size, as evidenced by the color change and spectral characteristics of the suspension (Figure 3). Thus, the increase in the size of AuNPs in the conjugate with streptavidin can be carried out in a wide pH range with a shift to the region of acidic values (from 3.0 to 6.0) compared to native nanoparticles.

### 3.4. Effect of pH on the AuNP Label Growth during Au^3+^ Reduction on Nitrocellulose Membrane

**Figure 4:**
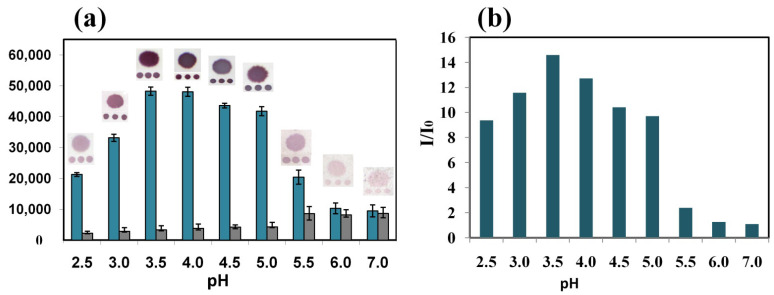
Absolute intensities of staining of specific spots (blue) and areas around the spots (gray) after AuNP enhancement at different pH values (**a**); signal-to-noise ratios at different pH values (**b**). Hybridization of 150 pM of target oligonucleotide.

Studying the enhancement of AuNP labels in membrane DNA-microarray technique with scanometric detection was performed with model oligonucleotides: capture and control oligonucleotide probes (Table S1) were immobilized on the microarrays fabricated on nitrocellulose membranes, and biotinylated target oligonucleotides of 19 bases, complementary to the capture probes, were hybridized with them. Then, biotin labels were revealed with streptavidin-AuNPs conjugate, and the enhancement procedure of HAuCl_4_ reduction with NH_2_OH was performed at different pH values. To prepare the enhancement solution, a different order of reagent addition was used in contrast to the standard one used earlier: the pH of the HAuCl_4_ solution was adjusted to a certain value; then it was mixed with hydroxylamine and the membranes were immersed with the final composition. The staining intensities of the spots were determined together with the intensities of nonspecific hybridization on the control spots and the background staining of the areas around each test spot. The intensities of specific hybridization and the background staining around the spots and on the control spots depended on pH (Figure 4a). Background staining was similar for the control spots and the areas around the specific spots; it was attributed to the unspecific nucleation and/or to the growth of nanoparticles from the conjugate nonspecifically bound to the membrane support. Background staining was insignificant in the pH range from 2.5 to 4.5 and increased at higher pH values. As a result, the dependence of the signal-background ratio on pH had a bell-shaped dependence with a maximum at pH 3.5 (Figure 4b).

**Figure 5:**
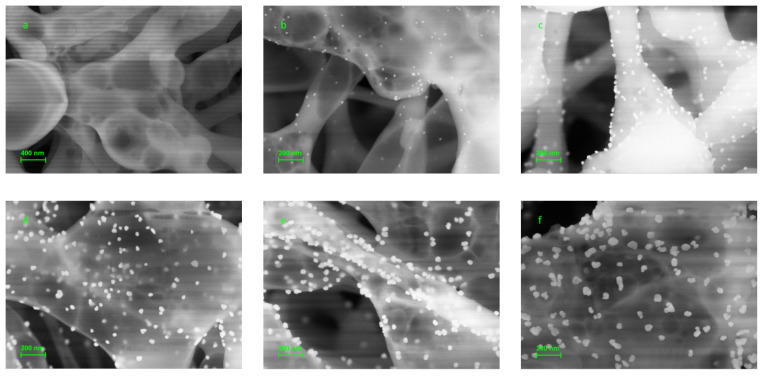
SEM images of the specific and control spots after hybridization of the membrane microarray with 150 pM of target oligonucleotide followed by enhancement procedure at pH range from 2.5 to 4.5. (**a**) Control spot; (**b**) specific spot without enhancement; (**c**–**f**) specific spot after enhancement at pH 2.5 (**c**); pH 3.0 (**d**); pH 3.5 (**e**); and pH 4.5 (**f**).

**Figure 6:**
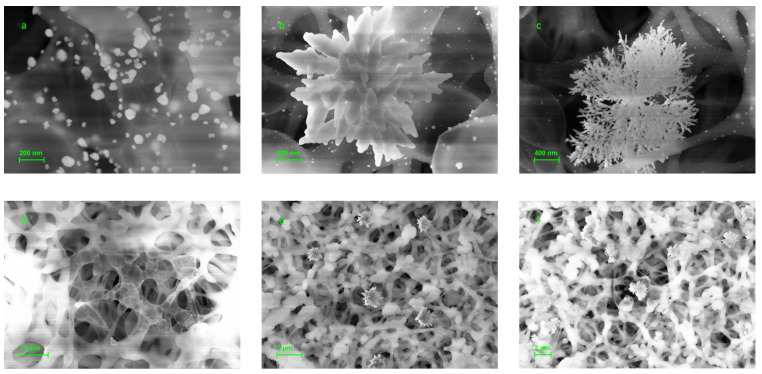
SEM images of the specific and control spots after hybridization of the membrane microarray with 150 pM of target oligonucleotide followed by enhancement procedure at pH range from 5.5 to 7.0. Specific (**a**) and control (**d**) spots after enhancement at pH 5.5; specific (**b**) and control (**e**) spots after enhancement at pH 6.0; specific (**c**) and control (**f**) spots after enhancement at pH 7.0.

Scanning electron microscopy was used to characterize the nanoparticles formed on the surface of the nitrocellulose support as a result of the enhancement reaction at different pH values. The results are shown in Figure 5 and Figure 6 for different pH ranges (below 5.0 and above 5.0), since the latter was characterized by a significant increase in the background staining. As can be seen from Figure 5a, there was minimal unspecific nucleation around specific spots in the pH range below 5.0, while nanoparticle labels were visible clearly on them (Figure 5b–f). It is obvious that the diameter of the AuNPs becomes larger after the enhancement procedure (Figure 5c–f) than before it (Figure 5b). Thus, reduction of HAuCl_4_ with NH_2_OH was specific in the pH range from 2.5 to 4.5: it led to diffuse growth of the existing AuNPs acting as seeds with autocatalytic properties and did not take place outside the specific spots. There was a clear tendency for larger particles at higher pH values of the enhancement solution (Figure 5c–f). The shape of the resulting AuNP aggregates was also pH dependent as was previously described in the literature [38,39,47]. The shape was spherical at pH values from 2.5 to 4, while at pH 4.5 particles were hardly spherical (Figure 5f). The formation of the most homogeneous particles of increased size (mean diameter of 134 ± 14 nm) was observed at pH 3.5, which was four times bigger than before the enhancement (33 ± 2 nm).

At a pH above 5.0, an irregular growth of AuNPs in the test spot was revealed (Figure 6); moreover, the formation of star-like and snowflake-like aggregates was observed. It was coupled with more intensive nucleation without diffuse growth of the existing nanoparticle seeds. Nucleation also occurred around the spots followed by enlargement of newly synthesized seeds increasing the background staining (Figure 6d–f). Thus, more acidic pH values such as 3.5 seem to be more proper for the enhancement procedure.

### 3.5. Optimization of the Ratio of Reagent Concentrations for AuNP Enhancement on the Membrane Support

**Figure 7:**
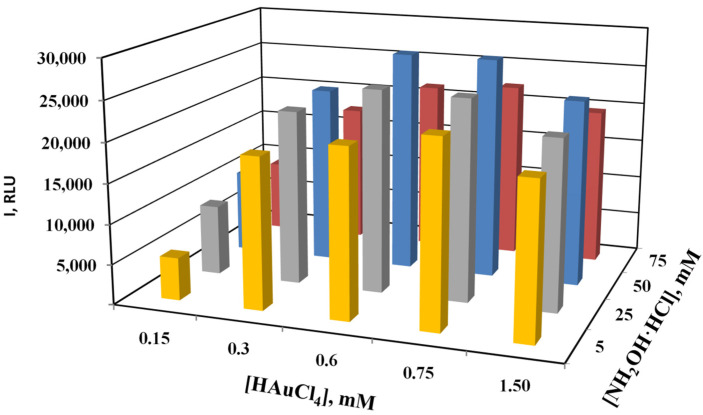
Diagram of the staining of specific spots on the concentration of reagents in the enhancement solution at pH 3.5.

In the literature, various ratios of reagents were used to increase the size of AuNPs in solution and on the surface of membrane carriers [39,48,50]. The ratio of reagents is important for enhancement since it helps to regulate the parallel processes of growth of the label size and the formation and growth of new seeds. Figure 7 shows the results of the reagent concentration optimization in the hybridization analysis of a short 19-mer oligonucleotide on a nitrocellulose membrane. The optimal concentrations for enlargement of the AuNPs on the membranes were 0.6–0.75 mM of HAuCl_4_ and 50 mM of NH_2_OH, which corresponds to their molar ratio from 60 to 80. These ratios are one order of magnitude higher than previously used in the literature. An increase in the concentration of hydroxylamine probably leads mainly to an increase in the rate of reduction of HAuCl_4_ on the AuNPs in the DNA duplexes and avoids the formation of new seeds and their growth and deposition on the membrane, increasing background staining.

**Figure 8:**
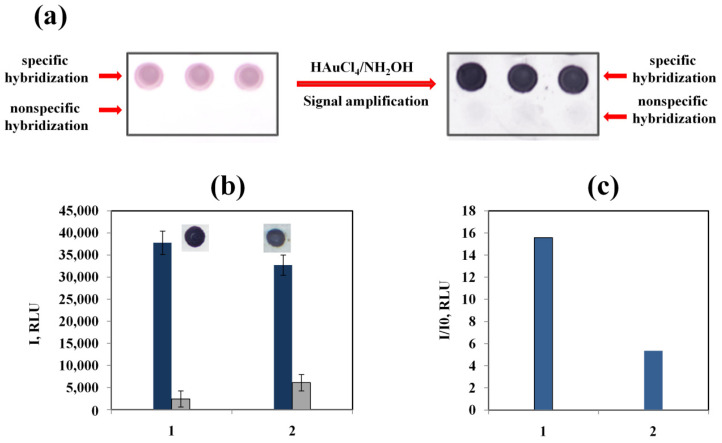
Images of nitrocellulose microarrays before and after the enhancement of AuNP labels using an enhancement solution at optimized reagent concentrations and pH (0.7 mM tetrachloroauric acid and 50 mM hydroxylamine, pH 3.5) (**a**); the staining intensities of specific (blue) and background (gray) spots with different orders of reagent mixing (**b**); and signal-to-noise ratios with different orders of reagent mixing (**c**). Target oligonucleotide (50 pM) was hybridized on the microarrays. 1—the pH value of tetrachloroauric acid solution was adjusted to 3.5 before the mixing with hydroxylamine; 2—the reagents were mixed without adjusting the pH value.

Figure 8 demonstrates the results of application of an enhancement solution at optimal pH and reagent concentrations when detecting a model oligonucleotide on membrane microarrays.

The use of large excesses of hydroxylamine and preliminary acidification of HAuCl_4_ solution made it possible to obtain the staining of specific spots of black color with low background staining (Figure 8a,b). Thus, under these conditions, a highly contrasting image of the result of specific hybridization is obtained, close to silver enhancement, without the occurrence of background reactions. The pH control of the tetrachloroauric acid solution makes it possible to increase the signal/background ratio by several times (Figure 8c), which improves the analytical performance of the method.

### 3.6. Seed-Mediated Growth of Nanoparticle Labels in Hybridization Membrane Analysis of DNA

**Figure 9:**
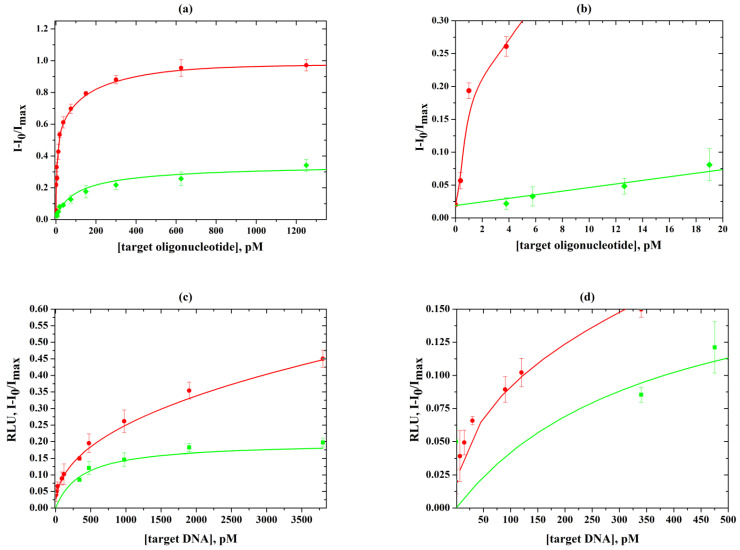
Calibration curves for the determination of short oligonucleotide of 19 bases (**a**) and long DNA of 860 bp, containing bla_TEM-1_ (**c**); enlarged sections of the calibration curves for low concentrations of short oligonucleotide (**b**) and long DNA (**d**). Green lines correspond to standard detection without enhancement; red lines correspond to the detection of AuNP labels with an enhancement solution at optimized concentrations and pH.

The optimized protocol of AuNP seed-mediated growth was applied for membrane microarrays with scanometric detection to improve the quantitative determination of a short single-strand oligonucleotide of 19 bases and long double-strand DNA of 860 bases, containing the gene of β-lactamase TEM-1 (Figure 9). DNA-target of bla_TEM-1_ was obtained by PCR from DNA fraction of a laboratory strain *E. coli*—producer of recombinant β-lactamase TEM-1. To characterize the increase in the sensitivity of the analysis, the LOD was determined as the mean signal intensity registered for a blank sample (0 pM DNA) plus two standard deviations. The lower LOD of 0.2 pM was determined for short oligonucleotides; it was almost two orders of magnitude lower compared to the LOD without the enhancement. When determining a long DNA molecule, enlargement of the size of the nanoparticles made it possible to reduce the LOD by one order of magnitude (35 pM) compared to the LOD without the enhancement.

**Figure 10:**
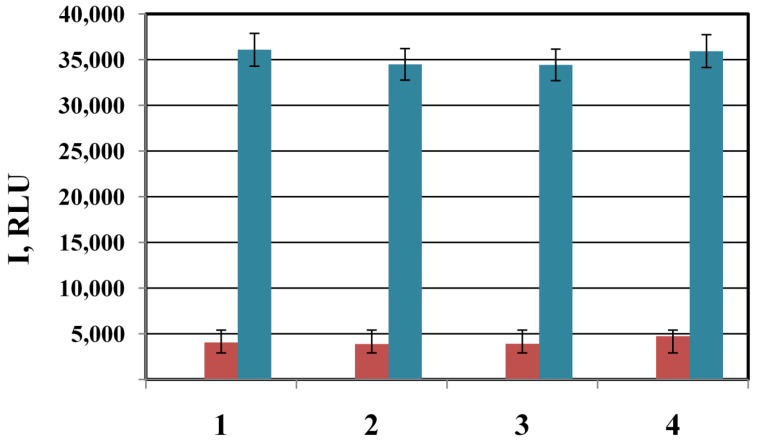
Determination of different target DNAs containing bla_TEM-1_ (1), bla_TEM-3_ (2), bla_TEM-37_ (3), bla_TEM-9_ (4) on nitrocellulose microarrays. Detection of AuNPs with an enhancement solution at optimized concentrations (blue); detection of AuNPs without enhancement (red). Concentration of target DNA 3 nM.

To demonstrate the practical applicability of the method, several samples of serine TEM-type β-lactamase genes isolated from Gram-negative bacteria resistant to β-lactams were used. The genes of this family are characterized by high homology and differ in several substitutions, leading to a change in the profile of the substrate specificity of the enzyme [51]. Figure 10 shows the results of the determination of four variants of β-lactamase genes (TEM-1, -3, -9, -37) by membrane hybridization analysis using AuNPs as labels. β-lactamase TEM-1 confer the bacterial resistance to penicillins and early cephalosporines; TEM-3 and -9 represent extended spectrum β-lactamases and confer the resistance to penicillins and cephalosporines of I-IV generations; β-lactamase TEM-37 confer the resistance to β-lactam inhibitors. To determine various TEM-type β-lactamase genes, we used an oligonucleotide probe corresponding to a conservative region of the gene sense sequence. All gene variants were successfully identified by the method. Seed-mediated growth of AuNPs under the optimized conditions made it possible to significantly increase (by 7–9 times) the intensity of staining spots. This demonstrates the advantages of the optimized protocol in improving the sensitivity of membrane-based analysis.

Table 2 presents the data on the quantitative determination of DNA by various methods, involving the microarrays on membrane supports, proposed recently. Most of them use AuNPs as labels for DNA duplexes, which are incorporated either directly into oligonucleotide probes or using the streptavidin-AuNP conjugate. Among the membrane-based methods, we demonstrated the lowest detection limit of a short oligonucleotide. There is a limited amount of evaluation of quantitative parameters for determining long DNA molecules in the literature. Therefore, we compared the detection limits of DNA when determining it on the microarrays of membranes and glass combined with scanometric detection, as well as using other methods with different supports and detection techniques.

The optimized protocol for the enlargement of the size of AuNP labels of membrane-based microarrays allowed us to significantly reduce the detection limit of DNA of a comparable size (genes of bacterial β-lactamases) when compared with glass-based microarrays with enzymatic detection and membrane-based chromatographic strips combined with recombinase polymerase amplification. The advantage of the proposed method is its applicability to different colorimetric membrane-based assays including the microarrays (for example, microarrays as components of devices for POC diagnostics) and chromatographic strips for express diagnostics that are combined with both instrumental and visual detection. The use of streptavidin conjugated with nanoparticles makes it possible to use a single universal approach for the multi-analyte analysis.

## 4. Conclusions

In this work, we performed a detailed investigation of the conditions for seed-mediated growth of AuNP labels in solution and on the porous nitrocellulose membranes widely used as a support for DNA detection by the microarrays and lateral flow analysis. The conditions of nanoparticle enlargement by the reduction of tetrachloroauric acid with hydroxylamine (pH values and concentrations of the reagents) were shown to strongly impact the resulting size/shape of AuNPs and their color intensity as well. The use of large excesses of hydroxylamine (one order of magnitude higher than previously used) and preliminary acidification of HAuCl_4_ solution results in highly contrasting staining of specific spots in black color without significant background reactions. Mean size of the resulting AuNPs was four times larger than before the enhancement. The DNA detection technique combined with the optimized protocol for AuNP label enlargement is characterized by enhanced sensitivity and high signal-to-noise values. The approach developed does not complicate the analysis and can be performed in a short period of time (about 2–3 min). The method showed an improved sensitivity for determining both short oligonucleotides and long DNA molecules using full-sized β-lactamase genes responsible for the development of bacterial resistance to β-lactam antibiotics. The optimized enhancement technique may be further transferred to other membrane-based colorimetric biosensors for DNA and RNA detection.

## Figures and Tables

**Table 1:** ζ-potentials (mV) of suspensions of gold nanoparticles and gold nanoparticles in a conjugate with streptavidin at different pH.

	pH Value	3.5	4.5	5.5	6.5	7.5
Object						
AuNPs		−22.4 ± 1.8	−32.5 ± 2.5	−43.2 ± 1.5	−45.8 ± 3.9	−50.6 ± 3.3
Conjugate Str−AuNPs		+28.3 ± 1.2	−1.6 ± 0.2	−22.5 ± 1.7	−30.8 ± 3.1	−37.8 ± 0.6

**Table 2:** Analytical performance of the methods developed for quantitative determination of DNA with different detection techniques, including enhancement of the nanoparticle labels.

Method/ Detection Principle	DNA Target Size	Label/ Enhancement	Limit of Detection (pM)	Reference
Determination of short oligonucleotides				
Hybridization on nitrocellulose microarrays/ Scanometric detection	Oligonucleotide (19 b)	Indirect labeling of DNA duplexes with AuNPs via streptavidin-biotin interaction/AuNP enhancement at optimized conditions	0.2	This work
Sandwich hybridization on the microelectrodes/Detection of conductivity	Oligonucleotide (27 b)	Direct labeling of detection oligonucleotide probe with AuNPs/ Silver enhancement	0.5	[52]
DNA hybridization with PNA probes/Colorimetric detection	Oligonucleotide (18 b)	Electrostatic interaction of DNA duplexes with AuNPs/ Gold enhancement	10	[53]
Hybridization on DNA microarrays/Scanometric detection	Oligonucleotide (21 b)	Labeling of the ds-DNA with DNA intercalator (daunorubicin) conjugated to AuNPs/ Gold enhancement	10	[54]
Sandwich hybridization on graphene-modified electrode/Differential pulse voltammetry	Oligonucleotide (30 b)	Direct labeling of a second capture oligonucleotide probe with AuNPs/Silver enhancement	72	[55]
Sandwich hybridization on plastic (polycarbonate) biochips)/Scanometric detection	Oligonucleotide (36 b)	Indirect labeling of DNA duplexes with AuNPs via streptavidin-biotin interaction/Silver enhancement	10,000 (10 nM)	[56]
Determination of long DNA				
Hybridization on nitrocellulose microarrays/ Scanometric detection	Full-size gene of β-lactamase bla_TEM-1_ (860 bp)	Indirect labeling of DNA duplexes with AuNPs via streptavidin-biotin interaction/AuNP enhancement at optimized conditions	35	This work
Hybridization on glass slides/Scanometric detection	Full-size gene of β-lactamase bla_CTX-M-5_ (870 bp)	Indirect labeling of DNA duplexes with horseradish peroxidase via streptavidin-biotin interaction	710 (0.40 ng μL^−1^)	[57]
Hybridization on membrane chromatographic strips combined with recombinase polymerase amplification/Colorimetric	Fragments of β-lactamase genes (bla_CTX-M_, bla_SHV_, and bla_OXA_) (296–593 bp)	Streptavidin-coatedblue latex	560–1100 (2.5 ng/25 μL)	[58]
Hybridization on the microarrays fabricated on silicon wafers/Detection of magnetoresistive ratio	Synthetic ssDNA (120 b)	Indirect labeling of DNA duplexes with magnetic NPs via streptavidin-biotin interaction	39	[59]
Adsorption of long DNA amplicons onto unmodified AuNPs prevents their salt-induced aggregation/Colorimetric	Fragment of *B. anthracis* genome (508 b)	Without labeling	10 pg	[60]

## Data Availability

Research data are not shared.

## Supplementary Materials

The following supporting information can be downloaded at: https://www.mdpi.com/article/10.3390/bios13010002/s1, Figure S1: SEM images of gold nanoparticles prepared by reduction of tetrachloroauric acid with sodium citrate; Table S1: Sequences of the oligonucleotides used in this work; Table S2: Characteristics of the spectra of suspensions of gold nanoparticles before and after the enhancement in solution; Table S3: Characteristics of the spectra of suspensions of gold nanoparticles in a conjugate with streptavidin before and after the enhancement in solution.

## Abbreviations

AuNP, gold nanoparticle; BSA: bovine serum albumin; EDC, 3-(3-dimethylaminopropyl)-1-ethyl-carbodiimide; EDTA, ethylenediaminetetraacetic acid; LFIA, Lateral Flow Immunochromatographic Assay; NHS, N-hydroxysuccinimide; SDS, sodium dodecyl sulfate; SEM, scanning electron microscopy.
