# Supplementary material for: Improvement of Seed-Mediated Growth of Gold Nanoparticle Labels for DNA Membrane-Based Assays
Source: Biosensors (Basel). 2022 Dec 21;13(1):2. doi: 10.3390/bios13010002 (PMC9855534; doi:10.3390/bios13010002)
Supplement: Supplementary file 1 [file biosensors-13-00002-s001.zip › biosensors-2030870-supplementary.pdf]

*Article*

# Improvement of Seed-Mediated Growth of Gold Nanoparticle Labels for DNA Membrane-Based Assays

**Galina V. Presnova [1], Gleb A. Zhdanov [1], Luibov Yu. Filatova [1], Mariya M. Ulyashova [1], Denis E. Presnov [2,3,4] and Maya Yu. Rubtsova [1,*]**

[1] Department of Chemistry, Lomonosov Moscow State University, 119991 Moscow, Russia
[2] D.V. Skobeltsyn Institute of Nuclear Physics, M.V. Lomonosov Moscow State University, 119991 Moscow, Russia
[3] MSU Quantum Technology Centre, 119991 Moscow, Russia
[4] Cryoelectronics Lab, Faculty of Physics, M.V. Lomonosov Moscow State University, 119991 Moscow, Russia
* Correspondence: mrubtsova@gmail.com

**Table S1.** Sequences of the oligonucleotides used in this work.

| Name | Sequence, 5’→3’ |
|---|---|
| **Capture oligonucleotide probe** | TTTTTTTTTTTTTTGATTGGACGAGTCAGGAGC |
| **Non-complementary oligonucleotide probe** | TTTTTTTTTTTTTTTCTAGACAGCCACTCATA |
| **Model target oligonucleotide** | Biotin-GCTCCTGACTCGTCCAATC |
| **Capture oligonucleotide probe for TEM type beta-lactamase** | TTTTTTTTTTTTTTGCTTCCCGGCAACAATTAATAGACTGGATGGAGGC |

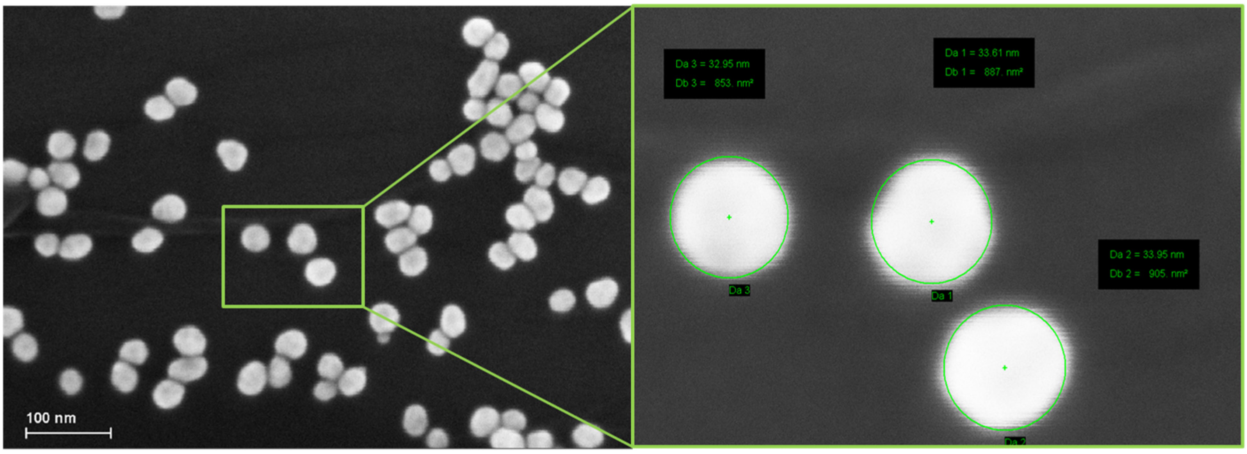


**Figure S1**. SEM images of gold nanoparticles prepared by reduction of tetrachloroauric acid with sodium citrate.

**Table S2.** Characteristics of the spectra of suspensions of gold nanoparticles before and after the enhancement in solution.

| pH value | Parameter | Without amplification | Reaction time | | |
|---|---|---|---|---|---|
| | | | 2 min | 5 min | 10 min |
| **2.0** | λ | 529 | 550 | 555 | - |
| | Peak value | 1.20 | 0.87 | 0.97 | 0.74 |
| | The area under the curve in the range of 400-700 nm | 173.73 | 234.68 | 263.66 | 203.99 |
| **3.0** | λ | 529 | 541 | 550 | 550 |
| | Peak value | 1.20 | 0.96 | 1.07 | 0.74 |
| | The area under the curve in the range of 400-700 nm | 173.73 | 242.89 | 286.13 | 208.82 |
| **3.5** | λ | 529 | 532 | 533 | 532 |
| | Peak value | 1.20 | 1.43 | 1.87 | 1.62 |
| | The area under the curve in the range of 400-700 nm | 173.73 | 265.41 | 345.76 | 299.76 |
| **4.0** | λ | 529 | 535 | 534 | 532 |
| | Peak value | 1.20 | 2.11 | 2.04 | 2.01 |
| | The area under the curve in the range of 400-700 nm | 173.73 | 353.42 | 354.87 | 348.96 |
| **5.0** | λ | 529 | 536 | 535 | 534 |
| | Peak value | 1.20 | 2.18 | 2.13 | 2.06 |
| | The area under the curve in the range of 400-700 nm | 173.73 | 364.29 | 389.15 | 390.28 |
| **6.0** | λ | 529 | 534 | 538 | 537 |
| | Peak value | 1.20 | 2.08 | 2.01 | 1.98 |
| | The area under the curve in the range of 400-700 nm | 173.73 | 386.94 | 416.33 | 415.96 |
| **7.0** | λ | 529 | 550 | 550 | 551 |
| | Peak value | 1.20 | 2.00 | 1.48 | 1.15 |
| | The area under the curve in the range of 400-700 nm | 173.73 | 444.19 | 358.09 | 295.63 |
| **8.0** | λ | 529 | 550 | 550 | 550 |
| | Peak value | 1.20 | 1.39 | 1.09 | 1.05 |
| | The area under the curve in the range of 400-700 nm | 173.73 | 354.26 | 295.02 | 279.96 |

**Table S3.** Characteristics of the spectra of suspensions of gold nanoparticles in a conjugate with streptavidin before and after the enhancement in solution.

| pH value | Parameter | Without amplification | | Amplification time | | |
|---|---|---|---|---|---|---|
| | | GNPs | Str-GNPs | 2 min | 5 min | 10min |
| 3.0 | λ | 529 | 531 | 536 | 548 | 548 |
| | Peak value | 1.20 | 1.32 | 1.27 | 1.58 | 1.84 |
| | The area under the curve in the range of 400-700 nm | 173.73 | 185.67 | 196.33 | 299.93 | 354.65 |
| 4.5 | λ | 529 | 531 | 546 | 546 | 546 |
| | Peak value | 1.20 | 1.32 | 1.19 | 1.51 | 1.81 |
| | The area under the curve in the range of 400-700 nm | 173.73 | 185.67 | 233.12 | 305.60 | 376.60 |
| 6.0 | λ | 529 | 531 | 556 | 556 | 556 |
| | Peak value | 1.20 | 1.32 | 1.54 | 1.72 | 1.77 |
| | The area under the curve in the range of 400-700 nm | 173.73 | 185.67 | 368.67 | 425.41 | 440.17 |
